# Burden of Comorbidities in Hospitalizations for Cannabis Use-associated Intractable Vomiting during Post-legalization Period

**DOI:** 10.7759/cureus.5502

**Published:** 2019-08-27

**Authors:** Sowmya Madireddy, Rikinkumar S Patel, Virendrasinh Ravat, Temitope Ajibawo, Anthony Lal, Jenil Patel, Riddhi Patel, Hemant Goyal

**Affiliations:** 1 Internal Medicine, Mamata Medical College, Khammam, IND; 2 Psychiatry, Griffin Memorial Hospital, Norman, USA; 3 Internal Medicine, Larkin Community Hospital, South Miami, USA; 4 Internal Medicine, Brookdale University Hospital and Medical Center, New York, USA; 5 Internal Medicine, Windsor University Medical School, Basseterre, KNA; 6 Epidemiology, University of Arkansas for Medical Sciences, Little Rock, USA; 7 Epidemiology, The University of Texas School of Public Health at Houston, Houston, USA; 8 Department of Gastroenterology & Hepatology, The Wright Center of Graduate Medical Education, Scranton, Pa, Scranton, USA

**Keywords:** marijuana, recreational marijuana, chronic marijuana abuse, cannabinoid hyperemesis syndrome, cannabinoids, vomiting, intractable vomiting, national trends, nis

## Abstract

Objective

The aim of this study was to observe the trends of intractable vomiting and cannabis use disorder (CUD) with demographic characteristics, medical and psychiatric comorbidities, and hospitalization outcomes.

Methods

We conducted a retrospective cohort study using the nationwide inpatient sample (2010 to 2014). Patients aged 16-50 years discharged with a primary diagnosis of intractable vomiting and CUD were included (N = 9,601). We used the linear-by-linear association chi-square test and independent-sample T-test for measuring the categorical and continuous data, respectively.

Results

The number of intractable vomiting hospitalizations with CUD had an increasing trend (*P *< 0.001) with a 28.6% increase over five years. About half of the study population included young (16-30 years, 48.4%) males (57.2%). There was a decreasing trend (P = 0.041) in the prevalence of intractable vomiting with CUD in non-Hispanic Whites and Blacks, whereas there was 778% increase in Hispanics. The mean length of stay was 3.2 days which had a decreasing linear trend, and total hospital charges showed an increasing trend (*P *< 0.001), averaging $22,890. Electrolyte disorders (55.3%), hypertension (25.3%), chronic lung disease (11.9%), and deficiency anemia (10.3%) constituted the majority of comorbidities, with anemia showing a statistically significant increasing trend (*P *= 0.004). Anxiety disorders increased from 20.8% to 30.8% over five years, whereas depression decreased from 19.2% to 16.4% (*P *< 0.001). Concomitant tobacco abuse/dependence was present in 41.2% of patients with CUD.

Conclusion

The results of our study show that the intractable vomiting hospitalizations related to CUD have increased significantly over a five-year period. The general public and healthcare practitioners should be made aware of the paradoxical gastrointestinal side effects of cannabis.

## Introduction

Cannabis use has been a major concern after its legalization in many states in the United States (US). As per the recent reports, about 24.6 million (8.9% of the population) were current cannabis users [1]. Cannabis use has increased majorly in males and unemployed individuals [2]. Intractable vomiting or hyperemesis has been shown to have a significant association with cannabis abuse or dependence due to which this area has been studied more frequently [3-4]. A nationwide study by Patel et al. found that cannabis abuse increases the risk of persistent vomiting-related hospitalization by 60.9% after controlling for confounding risk factors and comorbid substance use disorders [3]. 

Cannabinoid hyperemesis syndrome (CHS) includes continuous or cyclical episodes of nausea and vomiting that may be relieved by compulsive hot showers or bathing in cannabis users [5-6]. Although cannabis is Food and Drug Administration (FDA) approved for the use of chemotherapy-induced nausea and vomiting, physicians need to be careful with CHS in cannabis users presenting with intractable vomiting. Hence, it is important to study the trends of cannabis use-related intractable vomiting hospitalizations during the post-legalization period, and the effects of cannabis on gastrointestinal (GI) health [4].

Al-Shammari et al. found that cannabis use with intractable vomiting increased by 17.9% during 2009 (the year when medical cannabis laws were passed in many states in the US) as compared to the period of 1993-2008. The incidence rate of intractable vomiting rose by eight percent in the post-legalization period [7]. The increased cannabis use among the US population and its effects on the GI system are rising due to increased medical cannabis legalization and decriminalization of recreational cannabis use in many states in the US [6].

Thus, the purpose of our study was to utilize a nationwide inpatient data to observe the trends of intractable vomiting and cannabis use disorder (CUD) with demographic characteristics, medical and psychiatric comorbidities, and hospitalization outcomes including length of stay (LOS) and total charges.

## Materials and methods

Design and data source

We conducted a retrospective cohort study with the use of the healthcare cost and utilization project’s (HCUP) nationwide inpatient sample (NIS) database, sponsored by the agency for healthcare research and quality (AHRQ) [8]. The NIS is the largest publicly available inpatient database in the US which includes a 20% sample of all inpatient admissions from the non-federal hospitals in 45 states. The sample consists of an average of seven million discharges annually, representative of about 95% of the US national population [8]. Since there are no directly available international classification of diseases, ninth revision, clinical modification (ICD-9-CM) codes for CHS, we used the ICD-9-CM to identify patients discharged with a combined diagnosis of CUD with intractable vomiting in the NIS data from January 2010 to December 2014. This method has also been used in other published studies in the past to diagnose the patients with CHS [9].

Study population

Patients aged between 16 to 50 years who were discharged after an emergency department-based hospitalization with a primary diagnosis of CUD and other diagnoses of intractable vomiting were included in the analysis [7,9-10]. The cannabis abuse/dependence diagnoses were identified using the following ICD-9-CM codes: 304.30, 304.31, 304.32, 305.20, 305.21, and 305.22 [3,7,9,11]. Also, intractable vomiting was identified using ICD-9-CM code 536.2 [3,7,9]. Patients below 16 years and above 50 years of age were excluded to limit our analysis to the middle 90th percentile of the cannabis users in the US [3,12].

Variables

Included demographic variables were age, sex, race, and median household income, and primary payer/insurance. We calculated the length of stay (LOS) as the number of nights the patient was admitted in the hospital [13]. Total hospital charges were also analyzed; however, we would like to note that this charge does not include information about the professional fees and non-covered charges [13]. The comorbidities were defined as the co-occurring medical and psychiatric conditions. AHRQ comorbidity software was utilized to generate the binary variables [8].

Statistical analysis

We used the linear-by-linear association chi-square test and the independent sample T-test for measuring the categorical and continuous data respectively. The categorical variables were noted in the percentages. One-way analysis of variance (ANOVA) was used for continuous variables (LOS and charges) to measure the differences over the study period. We also used discharge weight as provided in the NIS data, to attain a nationally representative patient sample [8]. A *P*-value lesser than 0.05 was used depending on the sample size to determine the statistical significance of the tests. Statistical analysis in this study was performed using the statistical package for the social sciences (SPSS) version 25 (International Business Machines Corporation, Armonk, New York).

Ethical approval

The NIS is a publicly available de-identified database without any apparent patient's identity and clinical information. Therefore, the institutional review board (IRB) approval was not required for this study [8]. This dataset was also used in a past study by Patel et al. that targeted inpatients with CUD and did not require IRB approval [3,12].

## Results

We found that the number of intractable vomiting-related hospitalizations with CUD (total N = 9,601) had an increasing trend (*P* <0.001) from 824 (in 2010) to 3180 (in 2014), that is about 286% increase over a five-year period. The mean age of the cohort was 31.4 years. Interestingly, 48.4% of the study population was consists of younger individuals (16 to 30 years). The prevalence of intractable vomiting-related hospitalizations with CUD was found to be decreasing with age. Intractable vomiting with CUD was predominantly present in males (57.2%) and was a statistically non-significant non-linear trend of its prevalence with respect to age and sex over the five-year period. There was a decreasing trend (*P* = 0.041) in the prevalence of intractable vomiting with CUD in non-Hispanic Whites and Blacks, whereas there was 778% increase in Hispanics (N = 45 in 2010 and N = 395 in 2014). The proportion of intractable vomiting hospitalizations with CUD decreased with median household income above the 50th percentile, whereas there was a linear increasing trend in those from low-income families (*P* = 0.017) as shown in Table 1.

**Table 1:** Demographic trend, 2010-2014 The proportion between the years (2010-2014) was obtained using cross-tabulation and the linear-by-linear association chi-square test and was significant with a P-value ≤0.05 at 95%. confidence interval.

Variable	2010	2011	2012	2013	2014	Total	P-value
Age at time of admission, in %							
16-30 years	47.6	51.1	49.4	48.3	47.2	48.4	0.815
31-40 years	31.6	33.8	30.9	35.1	36.0	34.2	
41-50 years	20.9	15.1	19.7	16.7	16.8	17.4	
Sex, in %							
Male	57.6	58.2	57.5	57.0	56.6	57.2	0.328
Female	42.4	41.8	42.5	43.0	43.4	42.8	
Race, in %							
Non-Hispanic White	56.4	59.1	52.3	52.7	53.5	54.0	<0.001
Black	33.4	26.9	31.5	34.3	30.2	31.4	
Hispanic	6.1	7.5	11.7	9.0	13.0	10.4	
Other	4.2	6.5	4.5	4.1	3.3	4.2	
Median household income (percentile), in %							
0 – 25^th^ percentile	35.4	28.7	36.3	37.8	36.3	35.6	0.017
26^th^ – 50^th^ percentile	26.7	31.0	25.7	24.4	28.0	26.9	
51^st^ – 75^th^ percentile	22.8	26.0	25.1	23.6	20.9	23.2	
76^th^ – 100^th^ percentile	15.1	14.3	13.0	14.2	14.8	14.3	
Insurance, in %							
Medicare	7.4	15.1	10.2	12.4	10.2	11.2	<0.001
Medicaid	34.5	28.5	33.4	30.5	46.7	36.5	
Private	25.6	24.9	21.2	22.5	24.7	23.6	
Self-pay	23.1	25.6	27.3	25.4	15.7	22.4	
Other	9.4	5.9	7.8	9.1	2.7	6.3	

About one-third of the intractable vomiting-related hospitalizations with CUD were covered by Medicaid, with an increase of 42.5% from 2010 (N = 283) to 2014 (N = 1485). There was a significant linear decreasing trend observed in the uninsured population (self-pay and others) over the five-year period. The overall mean LOS was 3.2 days which had a decreasing linear trend, and total hospital charges showed an increasing trend (*P* <0.001), averaging $22,890.

Clinically associated comorbid conditions were assessed for trends using AHRQ comorbidity indicators. Fluid and electrolyte disorders (55.3%), hypertension (25.3%), chronic lung disease (11.9%), and deficiency anemia (10.3%) constituted the majority of these comorbidities, with anemia showing a statistically significant increasing trend (*P* = 0.004), whereas the presence of other comorbidities had a stable non-significant trend. Cardio-metabolic comorbidities including diabetes with chronic complications and obesity were less prevalent (5% to 6%) in patients which could be due to the relatively younger cohort. Nevertheless, these comorbidities showed a significant increasing trend (diabetes and obesity increased by 82.9% (N, 21 to 195) and 99.5% (N, 21 to 230); respectively) over the five years. We also evaluated the presence of psychiatric comorbidities in patients with intractable vomiting and CUD. Among psychiatric comorbidities, anxiety disorders increased from 20.8% to 30.8% over five years, whereas depression decreased from 19.2% to 16.4% (*P* <0.001). Concomitant tobacco abuse/dependence was present in 41.2% of patients. There was a statistically significant decreasing trend seen in the use of alcohol and amphetamine as shown in Table 2.

**Table 2:** Trend in comorbidities, 2010-2014 The proportion between the years (2010-2014) was obtained using cross-tabulation and the linear-by-linear association chi-square test and was significant with P-value ≤0.05 at 95% confidence interval.

Variable	2010	2011	2012	2013	2014	Total	P value
Medical comorbidities, in %							
Deficiency anemia	7.9	9.8	9.5	10.7	11.2	10.3	0.004
Chronic lung disease	11.2	11.1	11.8	12.6	11.8	11.9	0.393
Diabetes, uncomplicated	3.6	7.0	7.8	6.4	6.4	6.5	0.274
Diabetes with chronic complications	2.5	5.6	4.9	4.7	6.1	5.1	0.002
Hypertension	20.4	24.7	27.5	25.6	25.3	25.3	0.070
Fluid and electrolyte disorders	53.6	54.9	56.4	53.9	56.6	55.3	0.224
Renal failure	2.5	4.2	4.6	4.1	4.2	4.1	0.206
Obesity	2.5	3.7	7.5	5.6	7.2	6.0	<0.001
Psychiatric comorbidities, in %							
Depression	19.2	22.2	14.5	14.9	16.4	16.7	<0.001
Anxiety disorders	20.8	16.9	22.0	23.6	30.8	24.6	<0.001
Mood disorder	26.2	25.7	23.4	24.0	26.4	25.1	0.603
Schizophrenia and psychotic disorders	8.4	6.0	10.1	11.6	12.6	10.6	<0.001
Alcohol abuse/dependence	14.7	12.8	11.0	10.5	9.9	11.1	<0.001
Tobacco abuse/dependence	41.1	39.0	47.1	40.1	39.6	41.2	0.121
Opioid abuse/dependence	10.4	4.7	9.8	6.6	8.2	7.8	0.802
Amphetamine abuse/dependence	2.5	3.1	2.0	1.6	1.4	1.9	<0.001
Cocaine abuse/dependence	5.5	7.5	6.9	6.0	4.2	5.7	<0.001

## Discussion

The results of our study are significant because our analysis revealed that the number of intractable vomiting-related hospitalizations with CUD increased from 2010 to 2014. Our results are similar to a study conducted by Bollom et al. which showed that the rates of emergency visits in patients with vomiting with CUD increased from 2.3 to 13.3 per 100,000 with 68.5% increase in the inflation-adjusted associated cost [14]. This increase could be the result of an increase in the use of cannabis in the younger population or could be due to an increased awareness of CHS among healthcare providers.

In our analysis, there was a minor decrease in the intractable vomiting with CUD hospitalization trend in the non-Hispanic White and Black population with a significantly increasing trend noted among Hispanics over the five years. Many studies have shown that Black and Hispanic population has higher odds of cannabis dependence as compared to non-Hispanic Whites [3,15-17]. However, the information about the race-wise prevalence of intractable vomiting with CUD is almost non-existence. In a case series of 98 patients with CHS, 66 patients (80%) were reported to be Caucasian [18]. Similarly, our study also had a higher number of White as compared to other racial groups. These results indicate that non-Hispanic White population might be at higher risk of developing intractable vomiting with CUD as compared to other races.

Although there are no studies about the association between socioeconomic status (SES) and intractable vomiting with CUD and past studies, have shown conflicting data regarding cannabis use and patients’ SES. Some studies have found that cannabis use is higher among the population with high-income [19, 20] whereas some studies mention that low income is associated with higher cannabis use [21-22]. We report that a higher proportion of the patients with median household income between 26th and 50th percentile were admitted in the hospitals with intractable vomiting and CUD. More studies are needed to be done focusing on these conditions and SES. Also, we found that Medicaid insured the higher proportion of patients with CHS compared to other insurance (Medicare and private).

The pathophysiology of intractable vomiting with CUD remains unclear. There are two potential mechanisms which could explain the compulsive hot bathing behavior in CHS patients. First, the cannabinoid can cause a dose-dependent disequilibrium of the digestive and thermoregulatory system in the hypothalamus to which the brain reacts, making the patient take hot showers. Second, this behavior could be the result of an increase in the core body temperature coupled with a decrease in the skin temperature, which activated the hypothalamus, making the patient take compulsive hot showers [23].

The prevalence of anemia in patients with intractable vomiting and CUD was found to have an increasing trend from 2010 to 2014. We also found that alcohol use was significantly higher when compared to other substance use disorders among these patients. Alcohol use is also associated with various nutritional deficiencies such as iron, cobalamin, and folic acid, which increase the risk of development of anemia in this population [24]. Intractable vomiting associated with CUD may also add to the nutritional deficiencies and can worsen the anemia.

Moreover, cardiovascular risk factors such as diabetes and obesity showed a significantly increasing trend over the study period. Hyperemesis due to cannabis use can play a role in the higher risk of developing diabetic ketoacidosis in diabetic patients [25]. We also observed the increasing trend in the comorbidity of diabetes with complications in this study. According to a survey of two large nationally representative surveys of about 50,000 participants, it was found that the prevalence of obesity is lower in cannabis users as compared to non-cannabis users [26]. Our study found contrasting result regarding the comorbid condition of obesity, and it also showed an increasing trend over the years.

There was an increasing trend seen in the prevalence of psychiatric comorbidities such as depression, anxiety, schizophrenia, and psychotic disorders in patients with intractable vomiting and CUD. Several follow up studies have found a similar association, as mentioned by Rey and Tennant that further strengthens our findings among cannabis users [27]. Other substances such as amphetamine use had decreased while cocaine had a variable trend over the years. However, it was significantly associated with intractable vomiting and CUD. Overall, the hospitalization charge due to intractable vomiting with CUD has increased from 2010, indicating that cannabis use remains a significant burden on the healthcare system.

Further intractable vomiting cases with CUD need to be studied to understand the observed trend of increasing hospitalization charges despite a decrease in LOS, as seen in our study. As CHS or intractable vomiting associated with CUD can be fatal and lead to death in some instances [28]. So, it is crucial to study these conditions in detail and develop interventional strategies as cannabis cessation is the only cure for CHS [29-30].

The main limitation of this study is that due to the retrospective and administrative nature of the database. The information about the dose, route of ingestion, and duration of cannabis use were not available to better explain the pathophysiology. The other limitation of our study was that we might have overestimated the number of patients with intractable vomiting with CUD as other disease processes can also present with similar symptoms. Also, our sample population may have affected due to change during this period given increasing cannabis use and/or clinician awareness of CHS. However, the main strength of our study is a large number of patients in the database with the comparison of intractable vomiting-related hospitalizations with CUD in the nationally representative population.

## Conclusions

The use of cannabis is on the rise in the US with legalization for both medical and recreational purposes, and subsequently, it has also been increasingly seen in the inpatient population. Our study results show that the number of patients presenting to the hospitals with complaints of nausea and vomiting associated with CUD has been increasing significantly. It is sometimes difficult and time-consuming to diagnose the causes of persistent or intractable vomiting that can lead to costly, invasive procedures. Hence, we would like to advise that the healthcare practitioners should have a high index of suspicion for CHS in any patient who presents to them with intractable nausea and vomiting and history of chronic cannabis consumption or co-diagnosis of CUD. The results of our study add to the growing body of evidence to suggest for the need of prospective high‐quality studies on cannabis consumption and health outcomes. Both the patients and healthcare practitioners will need to carefully consider the anticipated benefits in light of potentially significant health risks.
